# 3D-Printed Filters for Efficient Heavy Metal Removal from Water Using PLA@CS/HAP Composites

**DOI:** 10.3390/polym15204144

**Published:** 2023-10-19

**Authors:** Yisu Wang, Yan Wang, Shuai Qiu, Chongyang Wang, Hong Zhang, Jing Guo, Shengfa Wang, Huixia Ma

**Affiliations:** 1 School of Textile and Material Engineering, Dalian Polytechnic University, Dalian 116034, China; tt19981228@163.com (Y.W.); 15542361271@163.com (S.Q.); wangchongyang_wcy@163.com (C.W.); zhang_hong1234@sina.com (H.Z.); guojing8161@163.com (J.G.); 2 DUT-RU International School of Information Science and Engineering, Dalian University of Technology, Dalian 116620, China; 3 Dalian Research Institute of Petroleum and Petrochemicals, Sinopec, Dalian 116045, China; mahuixia.fshy@sinopec.com

**Keywords:** chitosan, hydroxyapatite, 3D printing, adsorption

## Abstract

Chitosan/Hydroxyapatite composites, enriched with relatively active -NH_2_ and -OH groups, have emerged as promising adsorbents for heavy metal removal. In this study, we harnessed the potential of CS/HAP composites by developing monolithic PLA@CS/HAP filters utilizing 3D printing and freeze-drying techniques. These filters possess both macroscopic and microscopic porous structures, endowing them with exceptional capabilities for removing heavy metals from water. The adsorption properties of CS/HAP composites were explored by varying the dosage, duration, and initial concentrations of copper ions. The maximum adsorption capacity for Cu^2+^ was determined to be approximately 119+/−1 mg/g at the natural pH and 298 K. Notably, the monolithic PLA@CS/HAP filters demonstrated remarkable efficiency in the removal of copper ions, with 90% of copper ions effectively removed within a mere 2-h period in a cyclic adsorption experiment. Furthermore, the PLA@CS/HAP filters exhibited a robust dynamic Cu^2+^ removal capacity (80.8% or even better in less than 35 min) in a dynamic adsorption experiment. Importantly, all materials employed in this study were environmentally friendly. In summary, the PLA@CS/HAP filter offers advantages such as ease of preparation, eco-friendliness, versatility, and broad applicability in diverse wastewater treatment scenarios, thereby presenting a significant potential for practical implementation.

## 1. Introduction

The contamination of water with heavy metal ions has emerged as a critical global issue, given its detrimental impacts on both human health and the environment [1]. Among the various heavy metal ions, copper ions are particularly worrisome due to their widespread presence in industrial wastewater, agricultural runoff, and municipal wastewater. These ions are highly toxic and tend to accumulate in organisms, further amplifying their harmful effects. Consequently, the removal of heavy metal ions from water has garnered significant attention from researchers in recent years [2,3,4,5]. Over the past decades, several methods have been developed to address the removal of toxic heavy metal ions from wastewater, including chemical precipitation, ion exchange, membrane separation techniques, and adsorption [6,7,8,9]. However, many of these approaches suffer from limitations such as low removal efficiency and high energy consumption. Fortunately, adsorption has emerged as one of the most effective methods for removing heavy metal ions, particularly at low concentrations, in comparison to other techniques. This method offers several advantages, including high efficiency, eco-friendliness, low cost, and ease of operation [10,11,12,13,14,15].

Chitosan, as a natural polysaccharide, possesses remarkable adsorption properties [16]. It possesses functional groups such as -OH and -NH_2_, which can interact with copper ions, facilitating their adsorption in water [17]. Nevertheless, its practical applications have been limited by its poor mechanical performance. To overcome this challenge, researchers have explored the utilization of various materials to create composite adsorbents with chitosan, aiming to enhance its overall performance. Among these materials, hydroxyapatite stands out as an environmentally friendly option that can be easily synthesized, exhibiting an excellent capacity for removing heavy metal ions from water. It has been revealed that the adsorption of copper ions from wastewater by hydroxyapatite is attributed to a two-step mechanism involving surface complexation and exchange with calcium ions. In recent years, CS/HAP composites have been extensively studied and developed in various forms for the effective removal of heavy metal ions and organic dyes from water [18,19,20]. These composite materials comprising chitosan and hydroxyapatite demonstrate a significant potential for applications in the field of heavy metal removal from water.

In recent years, adsorbents have predominantly been prepared mostly in the form of powders and pellets. However, these adsorbents often confront challenges such as poor stability and difficulty in separation, significantly limiting their practical applications. In response to these challenges, researchers have shifted their focus toward developing novel adsorbents with improved properties for efficiently removing heavy metal ions from water. One approach involves introducing an adsorbent into a magnetic material, such as ferric tetroxide, and recovering the adsorbent using an applied magnetic field [21]. A series of chitosan/hydroxyapatite (CS/HAP) composites were prepared using the freeze-drying technique for the purpose of removing lead ions from water [22]. Another method entails the fabrication of bio-based thermoplastic polymer polycaprolactone (PCL)/succinic anhydride (SA) filaments by combining PCL with SA using a solvent, followed by inserting the resulting film into a hot extruder to create filaments [23]. The SA filaments are then used in 3D printing to produce scaffolds customized for the removal of copper ions from water. The resulting monolithic adsorbent material exhibits improved stability and ease of recovery, making it a more promising option for practical applications.

3D printing, as an emerging form of additive manufacturing technology, has gained significant attention among researchers due to its numerous advantages, including precise design capabilities and efficient manufacturing processes. Among the various 3D printing techniques, fused deposition modeling (FDM) stands out as a mature technology that is widely employed for producing monolithic adsorbent materials designed for the removal of heavy metal ions from water. Several approaches have been developed for the fabrication of monolithic adsorbent materials utilizing FDM technology. These methods involve the integration of the adsorbent material with a polymer matrix, which is subsequently processed into wire filaments for further manipulation using FDM. The first approach entails blending the adsorbent material with a polymer and extruding it into wire filaments, which are subsequently shaped using FDM techniques [23,24]. Alternatively, the second method consists of directly printing polymer wire filaments to create scaffolds, which are subsequently surface modified to incorporate a coating of the adsorbent material [25,26]. Finally, the third approach employs freeze-drying technology to inject a solution of the adsorbent material into the scaffold, followed by lyophilization to generate a monolithic adsorbent material [27]. However, the preparation method involving composite wire filament prints may lead to limited contact between the adsorption sites within the scaffold and the heavy metal ions present in the wastewater, potentially compromising the removal capacity. Furthermore, the complexity of modifying 3D printed scaffolds and subsequently coating them with adsorbents poses challenges in accurately determining the specific loading. This limitation hinders the investigation of the actual adsorption flux and impedes the study of methods to enhance the adsorption capacity. To address these limitations, the current study adopts a straightforward and efficient approach by combining FDM technology with freeze-drying to fabricate monolithic adsorbent materials.

In this study, a mixed solution of CS and HAP was injected into a specialized polylactic acid (PLA) scaffold, and a PLA@CS/HAP filtration system was designed and prepared with the use of freeze-drying and 3D printing technology. A Fourier transform infrared spectroscopy (FTIR), scanning electron microscopy (SEM), and energy dispersive spectroscopy (EDS) were used to analyze the properties of the CS/HAP composite adsorbent. The effects of contact time, initial concentration, and adsorbent quality on the ability of CS/HAP to remove copper ions from water were thoroughly examined and discussed. Furthermore, adsorption thermodynamics, isothermal adsorption curves, and adsorption kinetics were investigated. Detailed assessments of the mechanical properties and reusability of the PLA@CS/HAP composites were also conducted. The stable mechanical properties and micro and macro porous structures in the PLA@CS/HAP scaffold ensure exceptionally high removal efficiency through five recycling experiments. Additionally, we fabricated PLA@CS/HAP column filters to remove Cu^2+^ solutions from water under dynamic conditions, simulating real industrial applications. Leveraging the advantage of structure building through freeze-drying and 3D printing, as well as the abundant adsorption sites of CS and HAP, our research presents an innovative approach for the development of advanced adsorption materials. This method not only enhances the Cu^2+^ removal efficiency but also promotes the reusability and the biodegradability of the adsorbent material.

## 2. Materials and Methods

### 2.1. Chemicals and Reagents

Chitosan with a 95% degree of deacetylation was obtained from Sinoparm Chemical Reagent Corporation, Shanghai, China. Hydroxyapatite (nanopowder, <100 nm particle size (BET), ≥97%) was obtained from Aladdin Industrial Corporation, Shanghai, China. The PLA filament (Figure S1) was obtained from Suzhou Jufu Technology Co., Ltd. (Suzhou, China) with an approximate D-isomer content of 2%. All other reagents used in the experiments were analytical grade.

### 2.2. Preparation of Three-Dimensional PLA Scaffold and Column

The virtual models of the porous scaffold and column were created using a custom-written software called Cinema 4D (2021). Creality print software (V3.12.2) was used to build the 3D design file. Then the design file was transferred to Creality Ender-3 S1 Pro for printing. The printing parameters for the PLA scaffold and adsorption column (Figure S2) were set to a nozzle temperature of 200 °C and a bed temperature of 60 °C. The speed of the 3D printing was 50 mm/min for all models.

### 2.3. Preparation of the CS/HAP Composites & PLA@CS/HAP Filter

**Figure 1:**
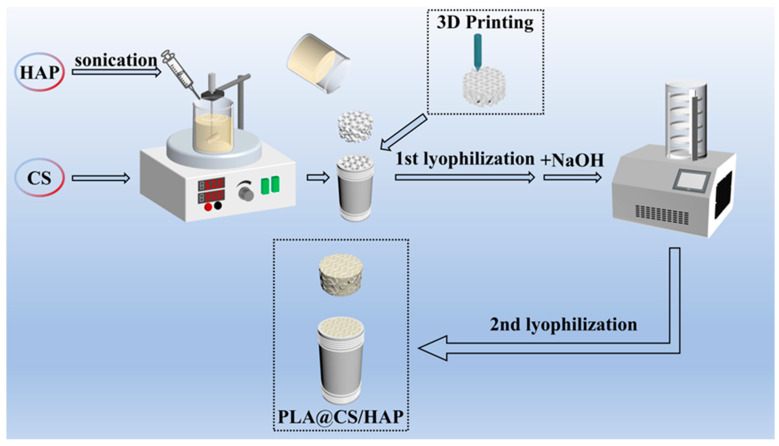
The schematic fabrication of the PLA@CS/HAP filter.

Chitosan (2.5 g) was dissolved in a 4% (*v*/*v*) acetic acid solution (50 mL) with continuous stirring. Separately, hydroxyapatite (2.5 g) was added to 50 mL of distilled water and subjected to sonication for 10 min. In this study, the 1:1 ratio (CS/HAP = 1/1) was employed in the preparation of the CS/HAP composite for all subsequent experiments (Figure S3). Then, hydroxyapatite was dispersed drop by drop into chitosan solution using a syringe. The mixture was agitated for 10 h until it was well mixed and became a viscous liquid. The mixed solution (100 mL) was transferred to the 3D-printed scaffold and was frozen at −80 °C for 12 h. Subsequently, the sample was first lyophilized at minus 50 °C using a freeze dryer to remove the solvent. Afterward, the CS/HAP composite was immersed in a NaOH (10%) solution for neutralization. Once neutralized, the sample was rinsed with distilled water and subjected to a second round of lyophilization. Finally, the CS/HAP composite was obtained. The synthesis process and fabrication of the PLA@CS/HAP filter is shown in Figure 1. To prepare the PLA@CS/HAP filter, the CS/HAP mixture was added into the PLA scaffold and column. The PLA@CS/HAP filter was prepared using the same process as the CS/HAP composite by freeze-drying twice. Finally, the PLA@CS/HAP filter was obtained.

### 2.4. Static Adsorption Experiments

Different concentrations of CuSO_4_ were used for static adsorption research. The degree of Cu^2+^ removal was tested by varying the adsorption time, adsorbent concentration, and initial Cu^2+^ concentration. CuSO_4_ solutions of different concentrations were prepared in beakers for use. The adsorbent and a 50 mL CuSO_4_ solution were mixed in a beaker to perform static adsorption in a shaker. The shaker was set to stir at 120 rpm at a temperature of 298 K. After static adsorption, the upper layer of CuSO_4_ was collected to calculate the adsorption capacity and removal efficiency using an Optima 8000 ICP-OES (Platinum Elmer, 940 Winter Street Waltham, MA 02451 USA). The equilibrium adsorption capacity (Qe) (Equation (1)) and the removal efficiency (R) (Equation (2)) were calculated as follows:

(1) $$Q_{e}=\frac{(C_{0}-C_{e})\times V}{m}$$

(2) $$R=\frac{\left(C_{0}-C_{e}\right)}{C_{0}}\times 100\%$$

where *C*_0_ (mg/L) and *C_e_* (mg/L) are the initial and final concentration of the adsorbed ion, respectively; *V* is the total volume of the solution (L); m is the mass of adsorbent (g).

In the reusability experiment, EDTA (0.5 mol/L) was used as the desorbing agents. A mixture of 25 mL of EDTA and PLA@CS/HAP filter (after adsorption) samples was allowed to react for 12 h at room temperature. Subsequently, the samples were washed with distilled water five times for each recycling cycle. The reusability experiment was repeated five times.

### 2.5. Cyclic & Dynamic Removal Experiments

**Figure 2:**
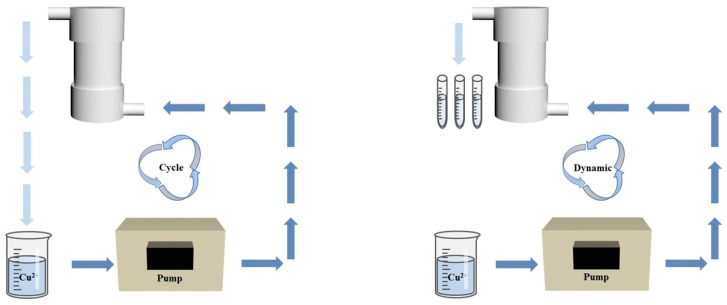
The schematic diagram of experimental setup for the cyclic & dynamic removal experiments.

To accelerate the removal of Cu^2+^, we conducted cyclic treatments using CuSO_4_ solutions, as depicted in Figure 2. The Cu^2+^ solution was pumped through a columnar filter at various flow rates (5 mL/min, 10 mL/min, 15 mL/min). The adsorption process was iteratively conducted through filtration cycles, and after each cycle, the Cu^2+^ concentration in the filtered solutions was analyzed using the ICP-OES to calculate the removal efficiency.

To simulate its application in industrial production, the treatment of the dynamic CuSO_4_ solution is shown in Figure 2. The sample (V = 15 mL, C_0_ = 300 mg/L) was passed through the column filter from top to bottom using a peristaltic pump. The solution was obtained from the outlet when the sample was exhausted, and the solution Cu^2+^ concentration was analyzed by the ICP-OES.

### 2.6. Structural and Morphological Characterization

The chemical structure was characterized by an FTIR (Elmer, Platinum, Waltham, MA, USA). The surface morphology of the CS/HAP composite was characterized using SEM (JSM6460LV, Japan Electronics Co., Ltd., Tokyo, Japan) with energy-dispersive spectroscopy (EDS) (X-MaxN, Oxford Instruments, Oxford, UK).

## 3. Results and Discussion

### 3.1. Characterization of CS/HAP Composites

**Figure 3:**
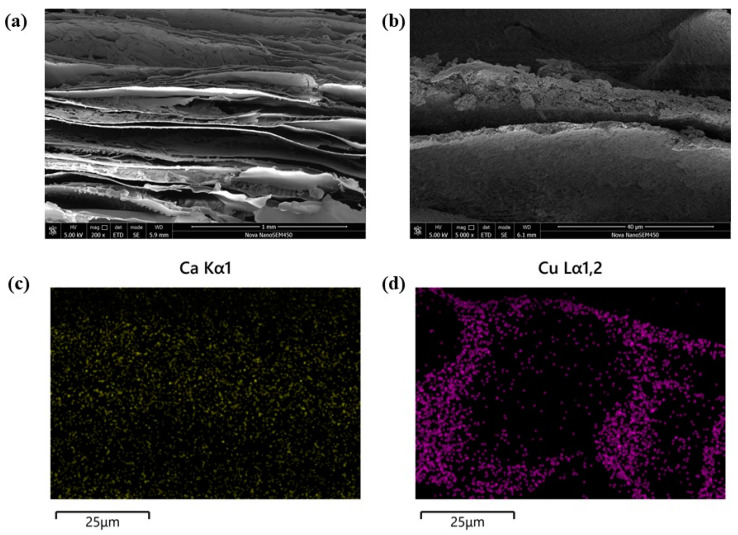
SEM of the CS/HAP composite: (**a**,**b**) before adsorption; (**c**) EDS images of Ca element distribution in CS/HAP composite before adsorption; and (**d**) Cu element distribution after adsorption, respectively.

**Figure 4:**
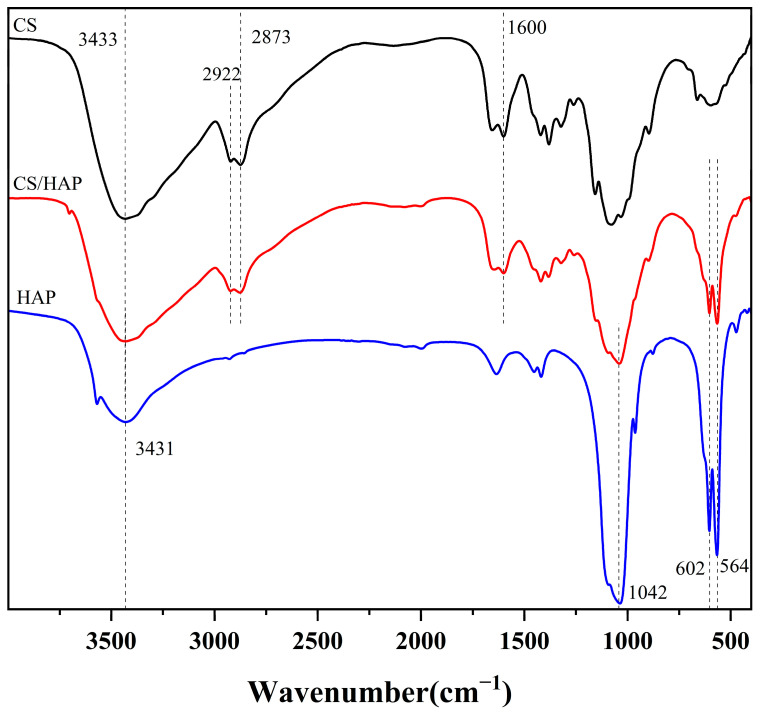
FTIR spectra of pure CS, HAP, and the CS/HAP composite.

The morphological characteristics of the CS/HAP composites were examined using SEM and EDS before and after the adsorption process. To enhance the removal of copper ions from water, the CS/HAP composites underwent freeze-drying, resulting in the creation of numerous channels. The electron micrographs (Figure 3) displayed no significant agglomeration, indicating that the preparation method, which involved the gradual addition of an HAP slurry into the CS solution following an ultrasonic dispersion procedure, facilitated the formation of composites with a uniform texture. Additionally, the distribution images of Cu elements after the adsorption process illustrated the successful adsorption of Cu ions onto the adsorbent. Furthermore, Figure 4 presents the FTIR spectra of pure HAP, pure CS, and the CS/HAP composite. The spectra of the CS/HAP composite exhibited characteristic peaks of both CS and HAP, with a slight shift in the band position and a reduced peak height. In the CS/HAP composite, the -OH of CS and HAP and -NH_2_ of CS stretching vibration bands were observed at 3433 cm^−1^, while the N-H bending vibrations appeared at 1600 cm^−1^. The C-H stretching vibrations were observed at 1922 and 1873 cm^−1^. The characteristic spectral bands of PO_4_^3−^ of HAP were observed at 1042, 602, and 564 cm^−1^. The FTIR data provided clear evidence of the formation of the CS/HAP composite.

### 3.2. Effect of Adsorbent Dosage

**Figure 5:**
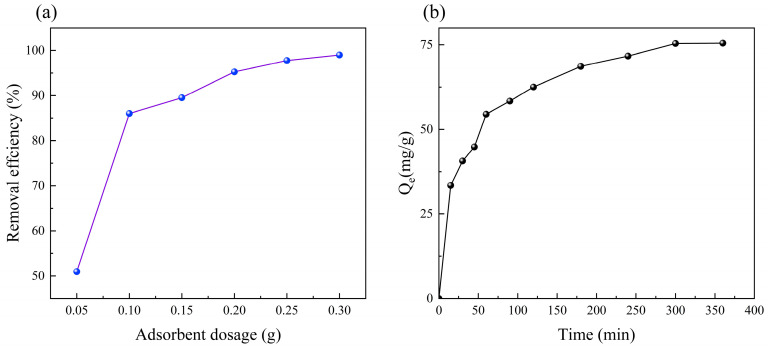
(**a**) Effect of adsorbent dosage and (**b**) time on the adsorption of Cu^2+^ by the CS/HAP composites.

The impact of the adsorbent dosage on the Cu^2+^ removal (initial concentration: 200 mg/L) was assessed within the range of 0.05–0.3 g (Figure 5a). Generally, augmenting the quantity of the sorbent, while keeping the time constant, led to an increased adsorption efficiency for Cu^2+^. Specifically, approximately 87% of the Cu^2+^ removal was achieved with 0.1 g of CS/HAP composites. As the adsorbent dosage increased to 0.3 g, the removal efficiency approached a high of 99%. Increasing the adsorbent dose from 0.05 g to 0.1 g increased the removal efficiency by 69%; however, increasing the adsorbent dose from 0.1 g to 0.15 g increased the removal efficiency by only less than 5%. Therefore, in the next investigation, an adsorbent dosage of 0.1 g was adopted.

### 3.3. Effect of Adsorption Time

The adsorption of Cu^2+^ on the CS/HAP composite was time dependent. In Figure 5b, the adsorption capacity increased rapidly to 54.48 mg/g in the first 60 min. It reached an equilibrium concentration of 75.40 mg/g at about 300 min. At the starting stage of the adsorption process, the high rate of adsorption was owed to the presence of a large number of adsorption sites on CS/HAP composite. Then, the adsorption rate became slower due to the reduction of available adsorption sites on the CS/HAP composite. In the slower stage, adsorption mainly occurred through the transfer of Cu^2+^ to the adsorption sites inside the adsorbent. After 300 min, the adsorption reached equilibrium. The high adsorption capacity of the CS/HAP composite is an ideal property for the removal of Cu^2+^ from water.

### 3.4. Effect of the Initial Cu^2+^ Concentration

**Figure 6:**
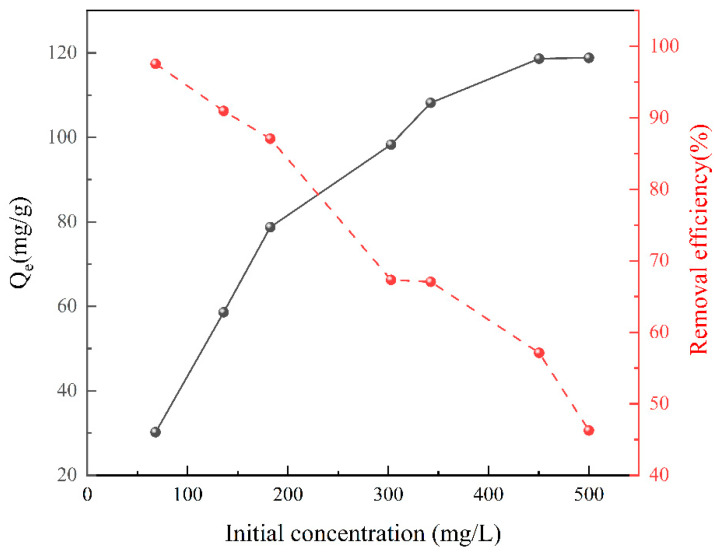
Effect of initial Cu^2+^ concentration on the adsorption of Cu^2+^ by the CS/HAP composites.

The influence of the initial Cu^2+^ concentration on the adsorption process was assessed, and the results are depicted in Figure 6. It was observed that the adsorption capacity consistently increased with a rise in the initial Cu^2+^ concentration. Up to an initial Cu^2+^ concentration of 450 mg/L, the low concentration of Cu^2+^ did not saturate all the available adsorption sites on the CS/HAP composite. Consequently, the adsorption capacity of the CS/HAP composite exhibited a significant increase with the augmentation of the initial Cu^2+^ concentration. In contrast, when the initial Cu^2+^ concentration was higher than 450 mg/L, there was almost no increase in the adsorption capacity due to the saturation of the adsorption sites of the CS/HAP composite [28]. The continuous decrease in removal efficiency was attributed to the decreasing fraction of Cu^2+^ that was removed from the solution.

### 3.5. Adsorption Isotherm

**Figure 7:**
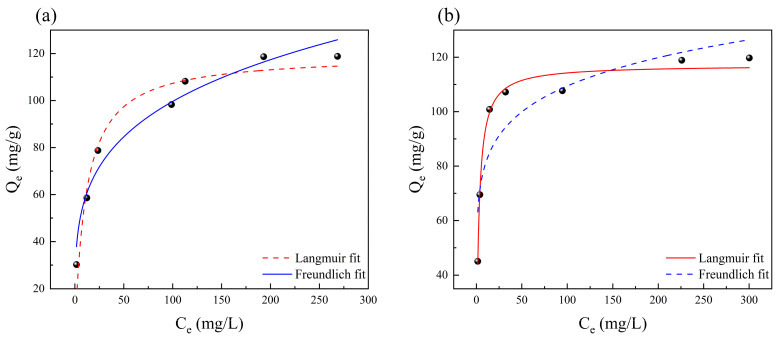
Adsorption isotherms for Cu^2+^ by the CS/HAP composites at (**a**) 298 K and (**b**) 318 K.

It is widely acknowledged that adsorption isotherm models play a pivotal role in accurately predicting the analytical and design outcomes of adsorption systems. In our experiment, the adsorption isotherm curve was meticulously acquired by varying the initial concentration of Cu^2+^, meticulously illustrated in Figure 7. To comprehensively analyze the experimental data, we applied two well-recognized models: the Langmuir and Freundlich models. The Langmuir isotherm model, in its essence, postulates a monolayer adsorption on a uniform surface without any intermolecular interaction. Conversely, the Freundlich isotherm model, an empirical equation, is adept at elucidating the heterogeneous adsorption process. These two models are expressed as follows:

Langmuir:

(3) $$Q_{e}=\frac{Q_{m}K_{L}C_{e}}{1+K_{L}C_{0}}$$

(4) $$R_{L}=\frac{1}{1+K_{L}C_{0}}$$

Freundlich:

(5) $$Q_{e}=K_{F}C_{e}^{\frac{1}{n}}$$

where *Q_e_* (mg/g) is the adsorption capacity at the adsorption equilibrium, *Q_m_* (mg/g) is the maximum adsorption capacity, *K_L_* (L/mg) is the Langmuir constant, *K_F_* (L/mg) is the Freundlich constant related to the adsorption capacity, and *n*^−1^ is the solvent parameter related to the adsorption strength.

In this study, we present the experimental data depicting the adsorption isotherm curves obtained by manipulating the initial Cu^2+^ concentration, along with their corresponding non-linear fits, displayed in Figure 7. The fitted results yield various values, which are systematically tabulated in Table 1. Notably, the correlation coefficient (R^2^) associated with the Freundlich model is observed to be much closer to 1, compared to that of the Langmuir model at 298 K. This discrepancy suggests that the Freundlich model aligns more suitably with the Cu^2+^ adsorption process. Conversely, at a higher temperature of 318 K, the Langmuir model provides a better fit to the Cu^2+^ adsorption process. Additionally, the Langmuir isothermal equation’s fundamental characteristics can be elucidated through the parameter R_L_ in Equation (4). Specifically, when RL falls within the range of 0–1, it signifies that the adsorption of Cu^2+^ can be considered as a process of “favorable adsorption”. At the same time, the Freundlich model is suitable for adsorption due to n^−1^ < 1 [29]. Overall, the adsorption of Cu^2+^ adheres to distinct adsorption models at varying temperatures. Particularly at elevated temperatures, the CS/HAP composites show a stronger inclination towards monolayer adsorption.

### 3.6. Adsorption Kinetics

To explore the potential rate-controlling step and adsorption mechanism for the Cu^2+^ removal, we employed both the pseudo-first-order and pseudo-second-order models for fitting analysis. The respective equations for these models are provided below. The pseudo-first-order model:

(6) $$Q_{t}=Q_{e}(1-e^{-k_{1}t})$$

pseudo-second-order model:

(7) $$Q_{t}=\frac{k_{2}Q_{e}^{2}t}{1+k_{2}q_{e}t}$$

where *Q_t_* (mg/g) is the adsorption capacity at time *t*, and *k*_1_ (min^−1^) and *k*_2_ (g mg^−1^ min^−1^) are the rate constants of the pseudo-first-order and pseudo-second-order models.

**Figure 8:**
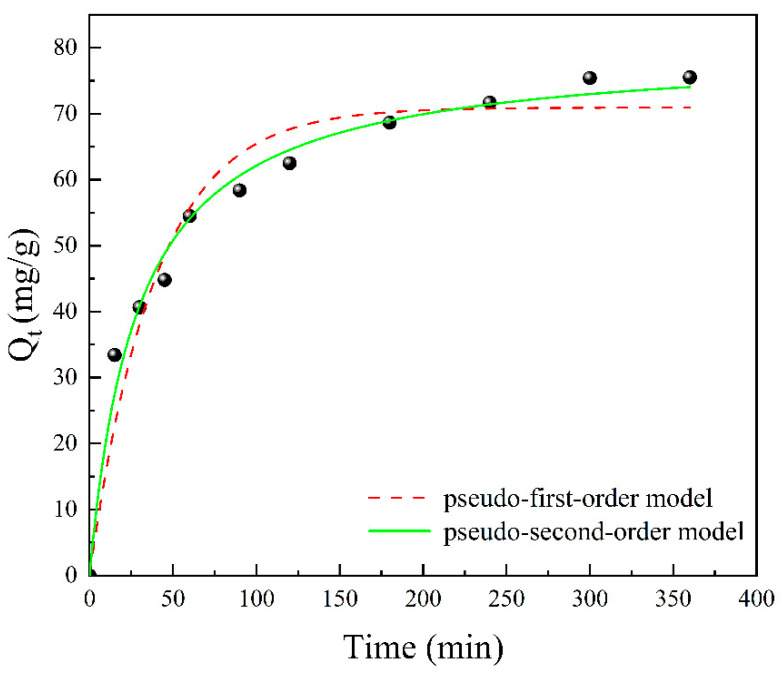
Adsorption kinetics of Cu^2+^ by the CS/HAP composites.

Figure 8 displays the adsorption kinetic curves observed for the CS/HAP composites at 298 K, and the corresponding adsorption kinetic parameters for these composites are summarized in Table 2. Notably, the correlation coefficient (R^2^) is found to be closest to 1 for the pseudo-second-order kinetic model. This observation signifies that the Cu^2+^ adsorption process aligns most closely with the pseudo-second-order kinetic model, which is well-suited for describing the chemical adsorption process [30].

### 3.7. Adsorption Thermodynamics

The adsorption temperature also affects the removal efficiency of Cu^2+^ by the CS/HAP composites (Figure S4). The removal efficiency of Cu^2+^ increased from 87.37% at 308 K to 93.18% at 318 K, indicating that adsorption is an endothermic process. With the increase in temperature, the molecular thermal motion becomes more pronounced, rendering the adsorption process more spontaneous and enhancing the collision between the adsorbent and Cu^2+^, consequently improving the removal efficiency. To analyze the thermodynamic process of the Cu^2+^ adsorption onto the CS/HAP composites, the following equations were employed to calculate the thermodynamic parameters:

(8) ΔG=ΔH−T×ΔS

(9) $$\Delta G=-R\times T\times ln(K_{d})$$

(10) $$K_{d}=\frac{K_{L}\times C_{ref}}{\gamma }$$

where Δ*G*, Δ*H*, and Δ*S* are the standard Gibbs free energy change (kJ mol^−1^), enthalpy change (kJ mol^−1^), and entropy change (J mol^−1^ K^−1^), respectively; R and T are the ideal gas constant (8.314 J mol^−1^ K^−1^) and Kelvin temperature (K), respectively. After converting K_L_ to units of (L/mol), K_d_ was calculated using Equation (10). The activity coefficient (γ) was determined using the Debye–Hückel and Davis relationships, depending on the ionic strength. Usually, the concentration of the solute (in the reference state) is considered equal to 1 M, i.e., C_ref_ = 1 mol/L [31,32,33].

As shown in Table 3, when the CS/HAP composites adsorb Cu^2+^, Δ*G* consistently appears negative at different temperatures, indicating that the Cu^2+^ adsorption behavior of the CS/HAP composites is spontaneous. Furthermore, the negative value increases with rising temperature, underscoring the increased spontaneity of the adsorption behavior at higher temperatures. A positive Δ*H* suggests that the adsorption process is endothermic, and elevated temperatures facilitate the forward progression of the adsorption process. The positive Δ*S* represents an increase in randomness at the solid–liquid interface during adsorption. Generally, when Δ*H* falls within the range of 20.9–418.4 kJ/mol, it indicates chemisorption due to the presence of strong chemical bonding [34]. Our results affirm that the adsorption behavior of Cu^2+^ on the CS/HAP composites exhibits chemisorption characteristics.

### 3.8. Properties of PLA@CS/HAP Filter

**Figure 9:**
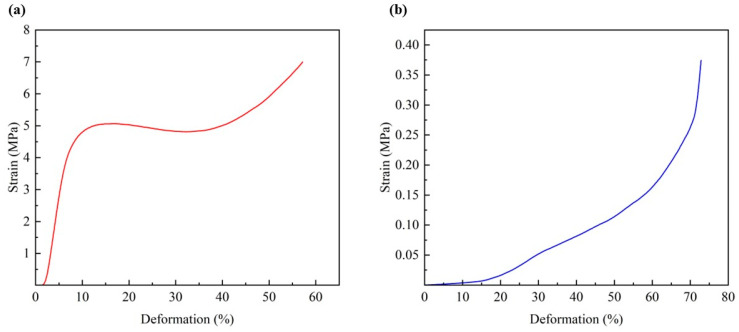
(**a**) Mechanical properties of the PLA@CS/HAP filter and (**b**) the CS/HAP composites.

**Figure 10:**
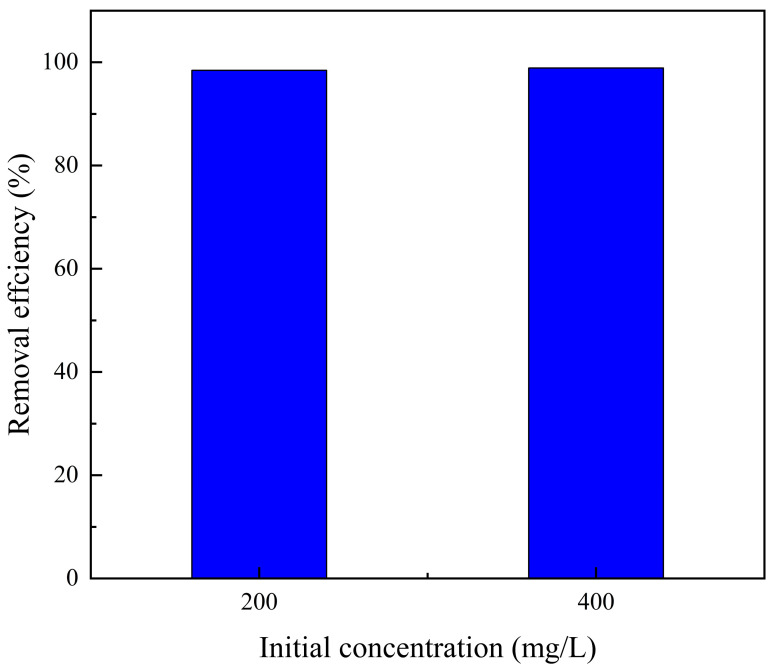
Removal efficiency of Cu^2+^ at two chosen initial concentrations of 200 and 400 mg/L.

Compression tests were conducted on the PLA@CS/HAP filters and CS/HAP composites to evaluate the mechanical properties of the adsorbed materials. The results revealed that the monolithic PLA@CS/HAP filter exhibited significantly enhanced compression resistance, thereby facilitating its storage and transportation shown as in Figure 9. Furthermore, in the context of the copper ion removal from water (Figure 10), the PLA@CS/HAP filter demonstrated an exceptional removal efficiency (>98%) for both lower initial concentrations (200 mg/L) and higher initial concentrations (400 mg/L). These compelling findings highlight the promising potential of the monolithic PLA@CS/HAP filter for the removal of copper ions from water, suggesting its favorable prospects in this field.

### 3.9. Reusability

**Figure 11:**
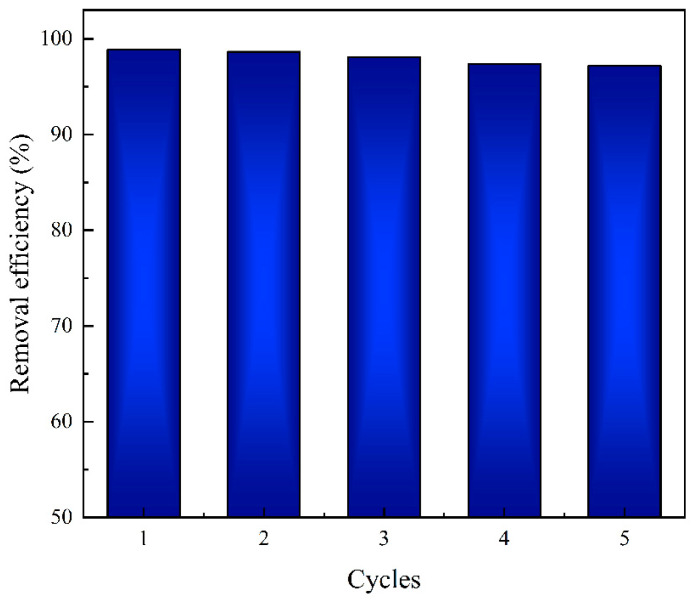
Adsorption reusability of Cu^2+^ by the PLA@CS/HAP filter.

In practical applications, the reusability of the adsorbent is of paramount importance. To assess the reusability of the PLA@CS/HAP filter, adsorption-desorption experiments were conducted. Figure 11 illustrates the reusability of the PLA@CS/HAP filter. Over five experiment cycles, the removal efficiency only slightly decreased from 98.9% to 97.17%. This minor reduction can be attributed to the abundance of adsorption sites on the PLA@CS/HAP filter, highlighting its stability.

Importantly, the physical appearance of the PLA@CS/HAP filter remained unchanged even after five cycles, underscoring its excellent reusability and stability. The comparison of the maximum adsorption capacity of Cu^2+^ by CS/HAP composite with other adsorbents is shown in Table S1. These findings demonstrate the promising potential of the PLA@CS/HAP filter for practical applications, making it a compelling choice due to its remarkable reusability and durability.

### 3.10. Cyclic & Dynamic Removal Experiment

**Figure 12:**
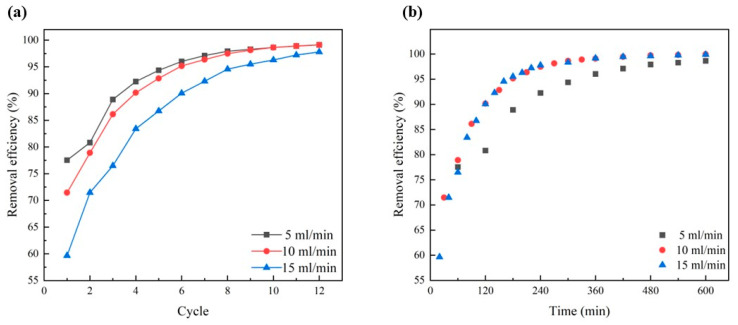
Effect of different flow rates on removal efficiency in cyclic removal experiment: (**a**) number of cycles and (**b**) time.

To enhance the removal of Cu^2+^ from the solution, PLA@CS/HAP columnar filters were created. A peristaltic pump was employed to pass 300 mL of solution through the columnar filter from bottom to top, improving the efficiency of the Cu^2+^ removal. The pump speed was adjusted (5 mL/min, 10 mL/min, 15 mL/min) to control the removal of the Cu^2+^ solution, and each cycle involved the volume of 300 mL of solution through the column filter. After each cycle, a certain amount of solution was taken to test the Cu^2+^ concentration. Figure 12 illustrates the excellent Cu^2+^ removal efficiency achieved at all three flow rates. Remarkably, the Cu^2+^ removal efficiencies of all three flow rates exceeded 90% after the sixth cycle, while the two flow rates of 5 mL/min and 10 mL/min achieved over 99% removal efficiencies after the twelfth cycle. Notably, the flow rate of 10 mL/min proved to be the most efficient for the removal of Cu^2+^ in this experiment. By the eighth cycle, the removal efficiency of Cu^2+^ at 10 mL/min equaled that of 5 mL/min. Subsequently, the removal efficiency of Cu^2+^ reached 99.95% after 10 h at a flow rate of 10 mL/min. Conversely, increasing the flow rate to 15 mL/min led to a slight decrease in the Cu^2+^ removal efficiency at each time stage. This decrease was attributed to the excessively fast flow rate, which hindered full contact between the Cu^2+^ solution and the column filter. In practical applications, precise control is crucial to ensure thorough contact between the copper ion waste solution and the monolithic PLA@CS/HAP columnar filter, thereby facilitating the effective removal of Cu^2+^. Consequently, the PLA@CS/HAP columnar filter demonstrates a significant potential for a wide range of applications in this field.

**Figure 13:**
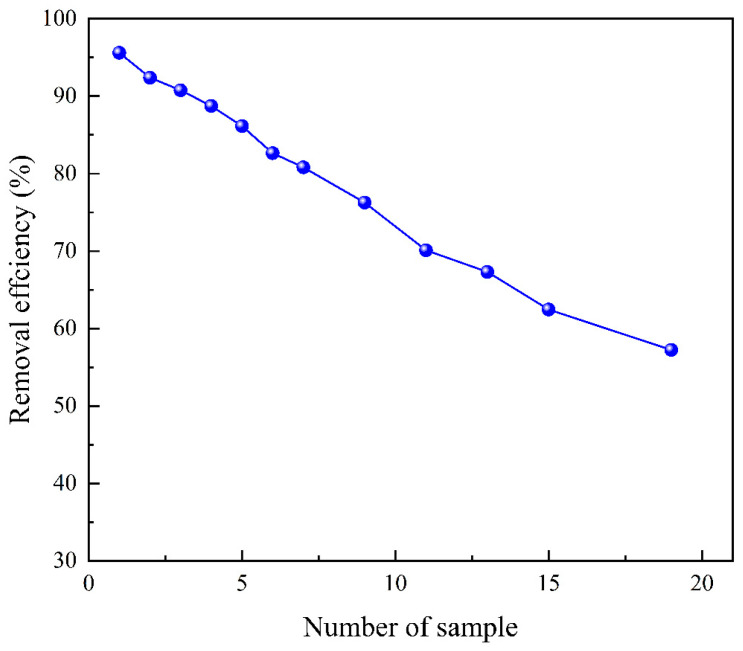
Removal efficiency of Cu^2+^ in dynamic removal experiment.

For the dynamic removal process, samples with identical volumes and initial Cu^2+^ concentrations were employed. The column filter exhibited a remarkable Cu^2+^ removal efficiency of 95.6% for the first sample as shown in Figure 13. Subsequently, for the seventh sample, the Cu^2+^ removal efficiency was 80.8% after passing through the column filter. Even after the fifteenth sample had been processed, the Cu^2+^ remained above 60%. Moreover, the dynamic Cu^2+^ removal time was dramatically reduced from 12 h to 55 min while maintaining the same removal efficiency. This substantial time reduction demonstrates the great potential for practical applications.

### 3.11. Removal Mechanism

**Figure 14:**
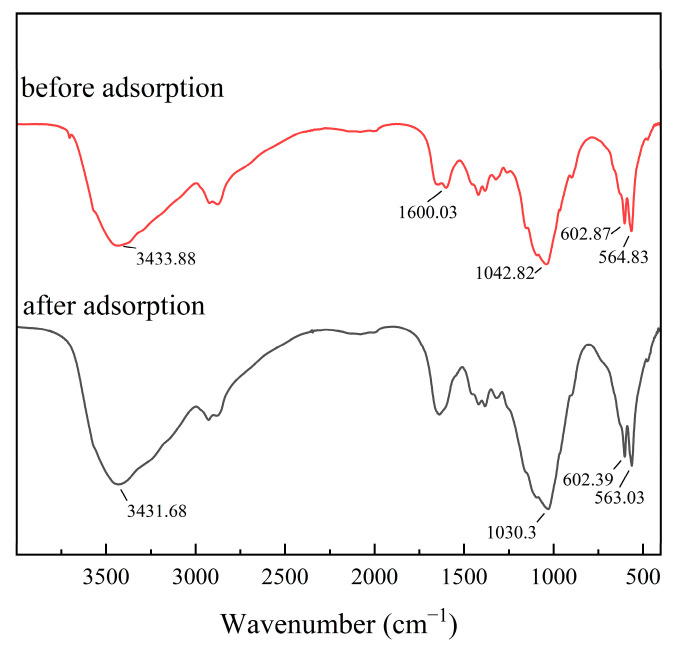
FTIR spectra of CS/HAP composite before and after Cu^2+^ adsorption.

**Figure 15:**
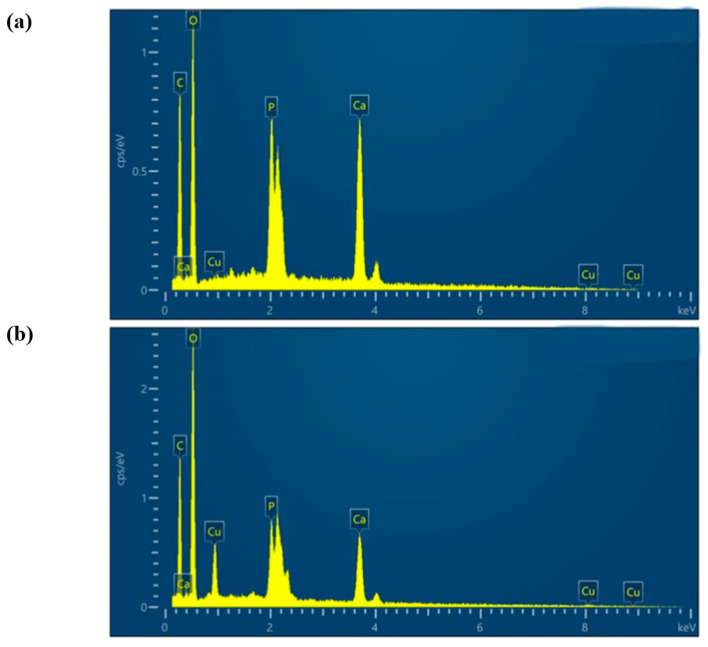
EDS images of CS/HAP composite before (**a**) and after (**b**) Cu^2+^ adsorption.

**Figure 16:**
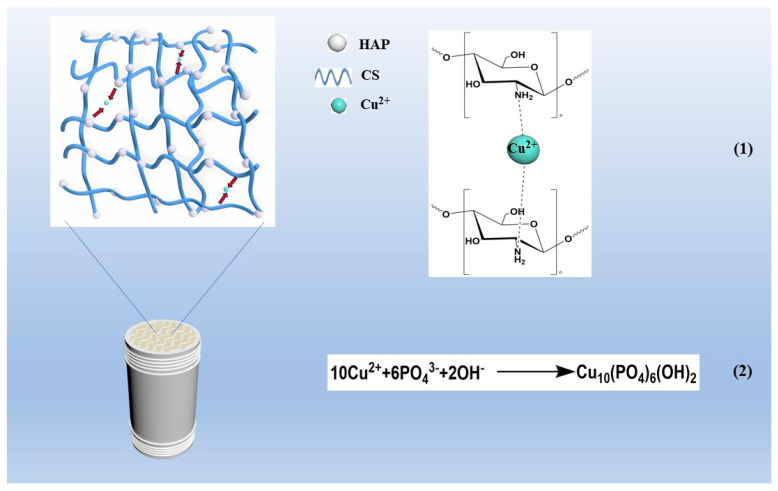
Mechanism diagram for the absorption of Cu^2+^ by PLA@CS/HAP filter.

To elucidate the adsorption mechanism, the adsorption process was analyzed using an FTIR and EDS. As shown in Figure 14, the adsorption bands corresponding to the stretching vibrations of the -OH and -NH_2_ groups in the CS/HAP composite, located at 3433.88 cm^−1^, experienced a shift to lower wave numbers after the adsorption of Cu^2+^ from the solution. Additionally, the characteristic spectral bands of PO_4_^3−^ (1042.82, 602.87, 564.83 cm^−1^) were also shifted after adsorption. Therefore, from the above results, -OH, -NH_2_, and PO_4_^3−^ groups are involved in the interaction of Cu^2+^ with the CS/HAP composites. Furthermore, as shown in Figure 15, the EDS elemental distribution map displayed a decrease in the proportion of calcium, while the proportion of copper increased from 0 to 9.1% in the energy spectrum. This observation suggests that the ion exchange took place during the adsorption process, involving the substitution of copper ions for calcium ions. Combined with the FTIR and EDS analysis, the adsorption mechanism is depicted in summary in Figure 16. It was concluded that the chelation of the chelating effect of the -NH_2_ and -OH groups in chitosan plays a crucial role in the adsorption of copper ions. Additionally, the exchange of Ca^2+^ ions from hydroxyapatite (HAP) with Cu^2+^ ions in the water leads to the formation of Cu-HAP, a phenomenon that has also been documented in other studies [19,22]. The monolithic PLA@CS/HAP filter demonstrates significant advantages in removing copper ions from water. Firstly, the incorporation of the 3D-printed PLA scaffold provides robust support, reinforcing the CS/HAP composite and ensuring structural integrity. Secondly, the PLA scaffold presents a macroscopic porous structure with interpenetrating pores, while the CS/HAP composite exhibits microscopically interconnected channels. This dual scale structure synergistically facilitates the efficient contact and adsorption of copper ions. Consequently, the filtration system exhibits an enhanced capacity for copper ion removal in water, thereby displaying promising prospects with broad applications.

## 4. Conclusions

The CS/HAP composite adsorbent and a series of monolithic PLA@CS/HAP filters were prepared using freeze-drying and 3D printing techniques. This cutting-edge method endowed the monolithic PLA@CS/HAP filters with remarkable macroscopic microchannel structures, facilitating their excellent Cu^2+^ removal capacity. The data fitting confirmed the conformance of the adsorption process to the Freundlich and pseudo-second-order models, indicating that the CS/HAP composite adsorbent underwent a multi-layer adsorption, chemisorption, which compounded the adsorption process. The Cu^2+^ reusability experiments demonstrated that the PLA@CS/HAP filters maintain a robust Cu^2+^ removal capacity even after undergoing five consecutive adsorption-desorption cycles with the removal efficiency remaining at 97.17%. Of special mention is the extraordinary efficiency of the integral PLA@CS/HAP filter in swiftly removing copper ions from water, achieving a high-speed copper ion removal of 80.8% in less than 35 min. All of the materials used in this study are environment-friendly, simple to prepare, easy to reuse, and have a low production cost, and therefore have a great potential for practical applications.

## Figures and Tables

**Table 1:** Fitting parameters for the adsorption isotherms of the CS/HAP composites towards Cu^2+^.

T (K)	Langmuir				Freundlich		
	Q_m_ (mg/g)	K_L_ (mg/L)	R^2^	R_L_	K_F_ (mg/g)	n^−1^	R^2^
298	119.498	0.0881	0.945	0.0314	33.529	0.2365	0.968
318	117.121	0.3954	0.985	0.0058	59.784	0.1313	0.828

**Table 2:** Fitting parameters for the CS/HAP composites’ adsorption kinetics.

T (K)	Pseudo-First-Order Model			Pseudo-Second-Order Model		
	Q_e_ (mg/g)	k_1_	R^2^	Q_e_ (mg/g)	k_2_	R^2^
298	70.93	0.02561	0.953	79.95	0.000436	0.986

**Table 3:** Thermodynamic parameters of the CS/HAP composites.

Temperature (K)	K_d_	Δ*G* (kJ mol^−1^)	Δ*H* (kJ mol^−1^)	Δ*S* (J mol^−1^ K^−1^)
298	6.739∙10^3^	−21.84	59.16	271.8
318	3.025∙10^4^	−27.28		

## Data Availability

The data presented in this study are available on request from the corresponding author. The data are not publicly available due to privacy.

## Supplementary Materials

The following supporting information can be downloaded at: https://www.mdpi.com/article/10.3390/polym15204144/s1, Figure S1. (a)TG of the 3D scaffold (PLA) and (b) DSC of 3D scaffold (PLA); Figure S2. photos of the PLA scaffold (a), PLA@CS/HAP(b), column PLA (c) and sorbent column PLA@CS/HAP(d); Figure S3. Effect of different chemical formulations on the adsorption of Cu2+ by the CS/HAP composite; Figure S4. Effect of temperature on the adsorption of Cu2+ by the CS/HAP composites, Table S1 Comparison of maximum adsorption capacity of Cu2+ by CS/HAP composite.
