# Supplementary material for: 3D-Printed Filters for Efficient Heavy Metal Removal from Water Using PLA@CS/HAP Composites
Source: Polymers (Basel). 2023 Oct 19;15(20):4144. doi: 10.3390/polym15204144 (PMC10610860; doi:10.3390/polym15204144)
Supplement: Supplementary file 1 [file polymers-15-04144-s001.zip › polymers-2655350-supplementary.pdf]

3D-Printed Filters for Efficient Heavy Metal Removal from Water Using PLA@CS/HAP Composites

Yisu Wang [1], Yan Wang [1,*], Shuai Qiu [1], Chongyang Wang [1], Hong Zhang [1], Jing Guo [1], Shengfa Wang [2,*] and Huixia Ma [3]

[1] School of Textile and Material Engineering, Dalian Polytechnic University, Dalian 116034, China; tt19981228@163.com (Y.W.); 15542361271@163.com (S.Q.); wangchongyang_wcy@163.com (C.W.); zhang_hong1234@sina.com (H.Z.); guojing8161@163.com (J.G.)
[2] DUT-RU International School of Information Science and Engineering, Dalian University of Technology, Dalian 116620, China
[3] Dalian Research Institute of Petroleum and Petrochemicals, Sinopec, Dalian 116045, China; mahuixia.fshy@sinopec.com
* Correspondence: wwangyan@163.com (Y.W.); sfwang@dlut.edu.cn (S.W.); Tel./Fax: +86-28-8632-3438 (Y.W.)

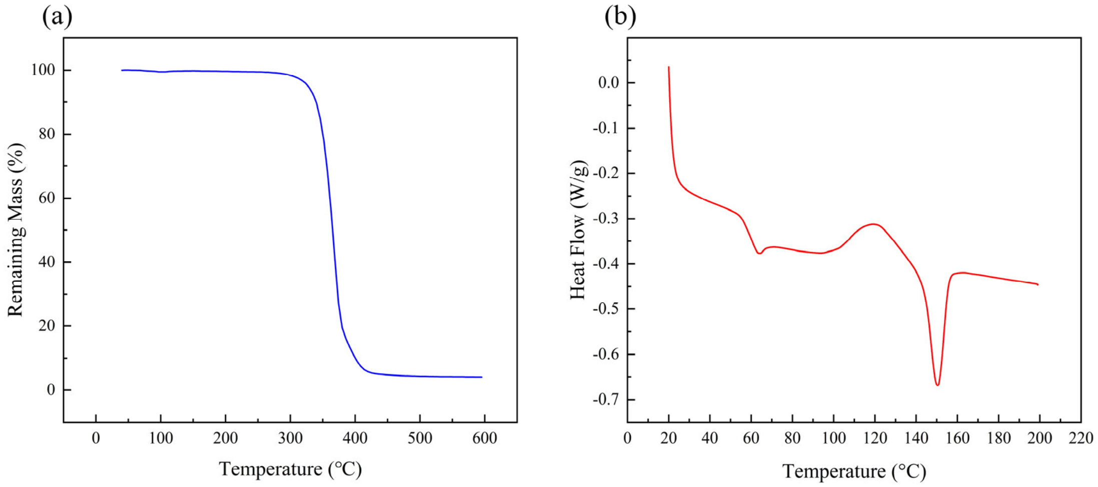

Figure S1. (a)TG of the 3D scaffold (PLA) and (b) DSC of 3D scaffold (PLA).

In this study, we employed a DSC-Q200 differential scanning calorimetry instrument (TGA2, METTLER TOLEDO, Switzerland) to conduct a thorough analysis of PLA's thermal behavior and crystallinity. During thermogravimetric analysis (TG), the impact of temperature escalation on polylactic acid (PLA) samples was observed. Within the initial temperature range (40°C to 130°C), the sample's mass remained

relatively stable, potentially influenced by volatile components. As temperature increased from 130°C to approximately 350°C, a noticeable reduction in mass, indicating a decomposition reaction was observed, with the decomposition rate accelerating with increasing temperature. Beyond 350°C, the mass loss gradually stabilized, implying the sample's transition into a steady state. Notably, a significant mass loss occurred around 350°C, suggesting the presence of a critical decomposition or reaction process. The glass transition temperature and melt temperature of PLA are measured at 60.9°C and 150.4°C, respectively. Additionally, the degree of crystallinity of PLA is found to be 2%.

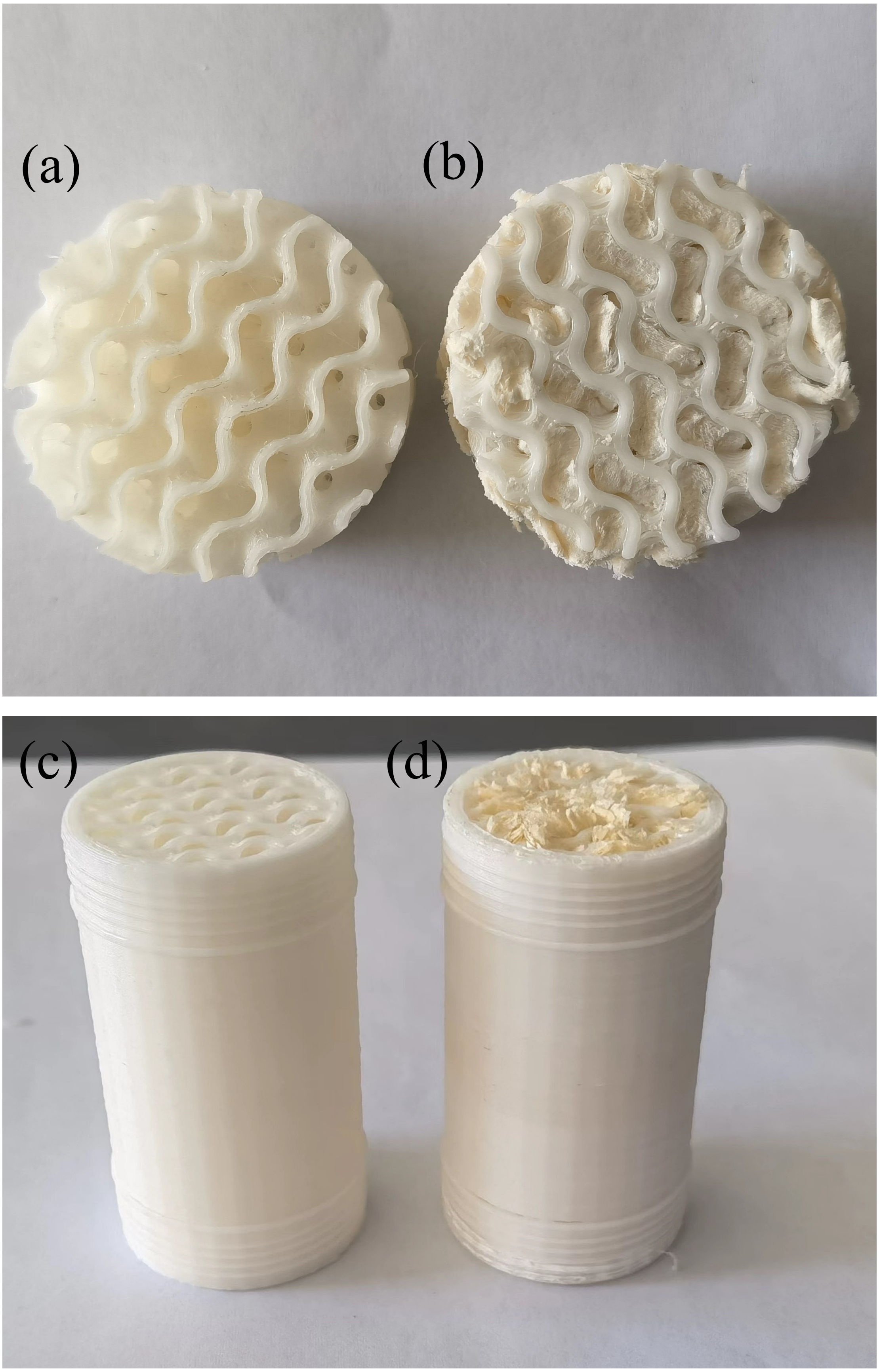

Figure S2. photos of the PLA scaffold (a), PLA@CS/HAP(b), column

PLA (c) and sorbent column PLA@CS/HAP(d).

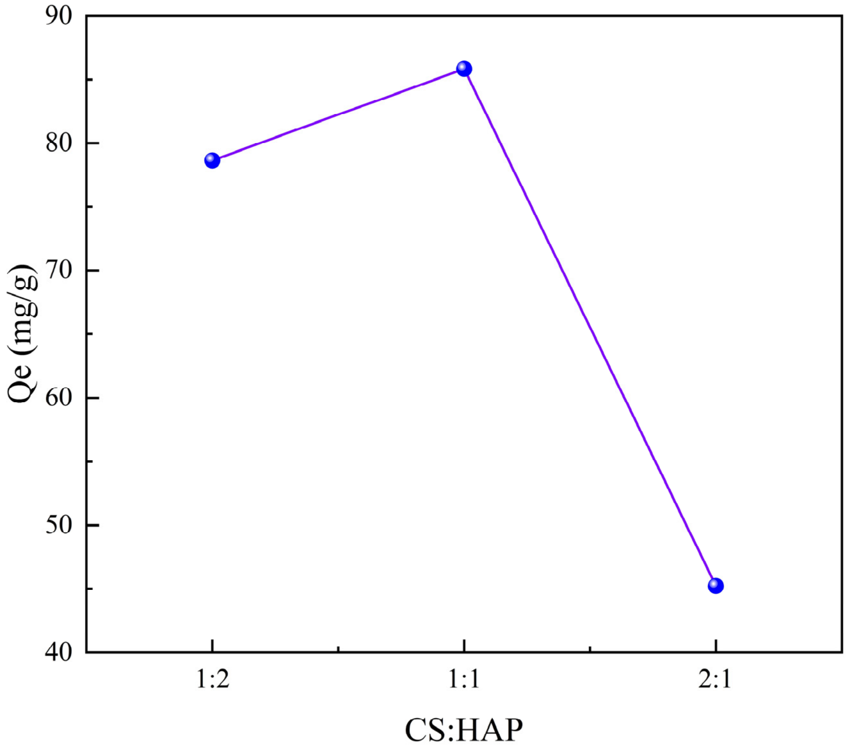

Figure S3. Effect of different chemical formulations on the adsorption of Cu^{2+} by the CS/HAP composite.

A set of three materials samples with different chemical formulations were used to removal Cu^{2+}. The adsorption capacity of them is shown in Figure. S3. The CS/HAP composite exhibits optimal copper ion removal capabilities from water at a 1:1 ratio of CS and HAP. Hence, this 1:1 ratio (CS/HAP=1/1) was employed in the preparation of the CS/HAP composite for all subsequent experiments.

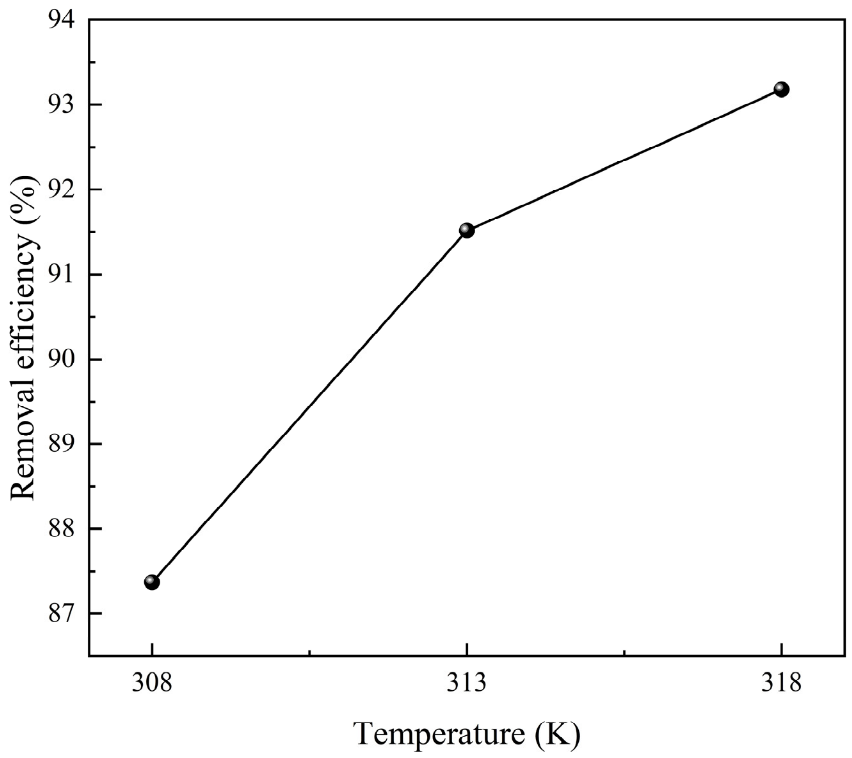

Figure S4. Effect of temperature on the adsorption of Cu^{2+} by the CS/HAP composites.

Table S1 Comparison of maximum adsorption capacity of Cu^{2+} by CS/HAP composite.

Adsorbent	Cu^{2+} (mg/g)	Reference
CS filter	13.7	(1)
MR-DPA	2.2	(2)
Carboxlated chitosan beads	86.0	(3)
Nano-hydroxyapatite/chitosan composite	26.11	(4)
Gelatin/zein/nHA nanofibrous membranes	67.8	(5)
CS/HAP composite	118.8	This work

1. Zhang D, Xiao J, Guo Q, Yang J. 3D-printed highly porous and reusable chitosan monoliths for Cu(II) removal. Journal of Materials Science. 2019;54(8):6728-41.

2. Villa-Reyna A-L, Aguilar-Martínez M, Ochoa-Terán A, Santacruz-Ortega H, Leyva-Peralta M-A, Vargas-Durazo J-T, et al. Efficient and Sustainable Bidentate Amines-Functionalized Resins for Removing Ag+, Cu2+, Pb2+, and Fe3+ from Water. Polymers. 2023;15(13).

3. Rajiv Gandhi M, Kousalya GN, Viswanathan N, Meenakshi S. Sorption behaviour of copper on chemically modified chitosan beads from aqueous solution. Carbohydrate Polymers. 2011;83(3):1082-7.

4. Rajiv Gandhi M, Kousalya GN, Meenakshi S. Removal of copper(II) using chitin/chitosan nano-hydroxyapatite composite. Int J Biol Macromol. 2011;48(1):119-24.

5. Deng L, Li Y, Zhang A, Zhang H. Nano-hydroxyapatite incorporated gelatin/zein nanofibrous membranes: Fabrication, characterization and copper adsorption. Int J Biol Macromol. 2020;154:1478-89.
